# The Risk of Systemic Diseases in Those with Psoriasis and Psoriatic Arthritis: From Mechanisms to Clinic

**DOI:** 10.3390/ijms21197041

**Published:** 2020-09-24

**Authors:** Yu Ri Woo, Chul Jong Park, Hoon Kang, Jung Eun Kim

**Affiliations:** 1 Department of Dermatology, Incheon St. Mary’s Hospital, College of Medicine, The Catholic University of Korea, Incheon 21431, Korea; w1206@naver.com; 2 Department of Dermatology, Bucheon St. Mary’s Hospital, College of Medicine, The Catholic University of Korea, Gyeonggi-do 14647, Korea; cjpark777smp@gmail.com; 3 Department of Dermatology, Eunpyeong St. Mary’s Hospital, College of Medicine, The Catholic University of Korea, Seoul 03312, Korea; johnkang@catholic.ac.kr

**Keywords:** psoriasis, comorbidity, cardiovascular disease, psoriatic arthritis, infection, cancer, sleep disorder, metabolic syndrome, autoimmune disease

## Abstract

Psoriasis and psoriatic arthritis (PsA) have been recently considered as chronic systemic inflammatory disorders. Over the past decades, enormous evidence indicates that patients with psoriasis and PsA have a higher risk of developing various comorbidities including cardiovascular disease, metabolic disease, cancers, infections, autoimmune disease, and psychiatric diseases. However, reported risks of some comorbidities in those with psoriasis and PsA are somewhat different according to the research design. Moreover, pathomechanisms underlying comorbidities of those with psoriasis and PsA remain poorly elucidated. The purpose of this review is to provide the most updated comprehensive view of the risk of systemic comorbidities in those with psoriasis and PsA. Molecular mechanisms associated with the development of various comorbidities in those with psoriasis and PsA are also reviewed based on recent laboratory and clinical investigations. Identifying the risk of systemic comorbidities and its associated pathomechanisms in those with psoriasis and PsA could provide a sufficient basis to use a multi-disciplinary approach for treating patients with psoriasis and PsA.

## 1. Introduction

Psoriasis is a common chronic immune-mediated inflammatory cutaneous disorder that affects about 0.91%–8.5% of the population [1]. Cutaneous manifestation of psoriasis can be characterized by well-demarcated erythematous thick scaly papules and plaques at distinct sites or disseminated over the whole skin. Such distinctive skin manifestations of psoriasis are due to disrupted differentiation and proliferation of keratinocytes resulting from interaction between altered immune cells and keratinocytes in a genetically-susceptible patient [2]. Besides the skin, similar inflammatory reactions can also occur at joints of susceptible patients and cause psoriatic arthritis (PsA), a form of arthritis. The diverse inflammatory molecules are released in skin lesions and joints of patients with psoriasis and PsA, and the vast majority of these molecules are also released into systemic circulation [3]. Indeed, the inflammation in those with psoriasis and PsA not only affects their skin and joints, but also spreads into their systemic circulation [3]. These findings provide a pathogenic basis for a possible association of various comorbidities with psoriasis and PsA.

In 1897, Strauss et al. [4] first reported the association between psoriasis and diabetes mellitus. Thereafter, various epidemiological studies have reported the association between psoriasis and possible comorbid diseases, including venous vascular disease such as thrombophlebitis, and venous thromboembolism and arterial vascular disease such as myocardial infarction, angina, and stroke [5,6,7,8]. Due to extensive methodological progress, many epidemiological studies have reported that the prevalence of various comorbidities is increased in those with psoriasis and PsA. Diverse disorders and their associated risk factors have also been identified. However, there remains an inconsistency among reported results regarding the risk of comorbidities in those with psoriasis and PsA.

Therefore, the aim of this study was to review the most up-to-date epidemiological data to identify the possible risk of various comorbid diseases in those with psoriasis and PsA. Among diverse comorbidities of those with psoriasis and PsA, this study focused on the risk of cardiovascular disease, metabolic syndrome, cancer, infection, autoimmune disorders, and psychiatric diseases in those with psoriasis and PsA in a more detailed manner. Moreover, this study intended to further analyze possible etiopathogenetic mechanisms of psoriasis and PsA and their comorbid disorders. Identifying the factual burden of comorbid disease in those with psoriasis and PsA might help us comprehensively manage patients with psoriasis and PsA.

## 2. The Risk of Cardiovascular Disease and Metabolic Syndrome in Psoriasis and Psoriatic Arthritis

Various studies have reported the association between cardiovascular disease (CVD) and psoriasis. In 2006, Gelfand et al. [9] performed a large population-based cohort study and found that severe psoriasis was an independent risk factor of myocardial infarction (MI). Using prospective data from the United Kingdom General Practice Research Database, they found that the incidence of MI per 1000 person-years was 3.58 (95% confidence interval (CI), 3.52–3.65) for healthy controls, 4.04 (95% CI, 3.88–4.21) for patients with mild psoriasis, and 5.13 (95% CI, 4.22–6.17) for patients with severe psoriasis [9]. They also revealed that the adjusted relative risk (RR) for MI was 1.25 (95% CI: 1.14–1.46) for 30-year-old patients with mild psoriasis and 3.10 (95% CI: 1.98–4.86) for 30-year-old patients with severe psoriasis. The adjusted RR for MI was 1.08 (95% CI: 1.03–1.16) for 60-year-old patients with mild psoriasis and 1.36 (95% CI: 1.13–1.64) for 60-year-old patients with severe psoriasis. These results imply that younger patients with severe psoriasis have a higher risk of MI than older patients. This study instigated various researchers to further identify the risk of CVD in psoriasis. Consequently, various epidemiological studies have reported that psoriasis is an independent risk factor for major adverse cardiovascular events (MACEs) including MI, stroke, and death caused by CVD. Although some studies have not found a statistically significant association between psoriasis and MACE [10,11,12,13], the majority of systematic review and meta-analysis have consistently shown significant associations between diverse CVDs and psoriasis [14,15,16,17,18,19,20,21]. Results from systematic reviews and meta-analyses assessing the association between CVD and psoriasis are summarized in Table 1.

Among them, Samarasekera et al. [18] and Armstrong et al. [19] both stratified the risk of CVD considering the severity of psoriasis and observed a higher risk of CV mortality, MI, and stroke in psoriasis patients with more severe disease. Concerning disease duration, Egeberg et al. [22] found that a longer disease duration had a stronger association with the risk of MACE (OR: 1.01; 95% CI: 1.00–1.01) than a shorter disease duration.

In addition, an emerging body of evidence suggests that treating psoriasis resulted in a decreased risk of CVDs. A study conducted in the USA concluded that treatment with methotrexate resulted in a decreased risk of MI in psoriasis than patients receiving topical therapy [24]. Recently, a variety of biologics have been used in managing psoriasis. The effects of biological therapies on CVDs of patients with psoriasis are currently receiving much interest. Of particular, psoriasis patients that received tumor necrosis factor (TNF) inhibitor showed a decreased carotid intima-media thickness [25], suggesting the protective effect of TNF inhibitors on CV risk in patients with psoriasis. In addition, the result from meta-analyses have found that using biologics such as interleukin (IL)-12/23p40 and IL-17A inhibitors in psoriasis was not associated with increasing the risk of MACEs [26,27]. Nonetheless, as role of IL-17 on development of atherosclerosis is still controversial, the results from IL-17 inhibitors on CV risk should be interpreted carefully.

The prevalence of CVD risk factors including hypertension, diabetes mellitus, dyslipidemia, and obesity was increased in patients with psoriasis. The combination of hypertension, central obesity, insulin resistance, and dyslipidemia, which is considered a metabolic syndrome, is also associated with the psoriasis. The recent findings from the systematic review and meta-analysis about risk of CVD risk factor in psoriasis are summarized in Table 2.

With regards to PsA, a recent meta-analysis assessing the risk of CV events in patients with PsA has shown that patients with PsA also have increased risk of CV events (OR: 1.43; 95% CI: 1.24–1.66) than the general population [23]. Furthermore, patients with PsA have 43% of increased CVD and 22% of increased cerebrovascular morbidity than the general population [23], presenting an obvious link between PsA and CV events.

Although various epidemiological studies have also identified the increased risk of CVD risk factors in patients with PsA [40,41,42], the meta-analysis comprehensively assessing the risk of CV risk factors in PsA has been sparse to date. Among various CVD risk factors, a study by Coto-Segura et al. [31] only concluded that patients with PsA showed the increased risk of type II diabetes mellitus (OR: 2.18; 95% CI: 1.36–3.50) than controls in their meta-analysis.

Pathophysiological mechanisms involved in the association of CVD with psoriasis and PsA can be explained by multifactorial etiologies including genetics, T helper (Th)1- and Th17-pathways, neutrophils, angiogenesis, and dysfunctions of endothelial cells and adipose tissues [43,44,45,46].

Although Koch et al. [47] did not find a possible association between psoriasis and cardiometabolic traits on a genetic basis, other researchers observed altered expression of genes related to metabolic disease and CVD in the skin lesions and sera of patients with psoriasis [48,49,50]. Eiris et al. [48] have found significant associations of single nucleotide polymorphism genotypes including *IL12B rs6887695-CC*, *IL12B rs3212227-CC*, and *IL23R rs2201841-GG* with psoriasis and type 2 diabetes. Among targeted artherosclerotic CV disease genes, monocyte chemoattractant protein-1 and macrophage-derived chemokine showed increased expression, while liver X receptor-alpha and peroxisome proliferator-activated receptor-alpha showed decreased expression in skin lesions of patients with psoriasis [49].

Psoriasis has long been known as a T-cell mediated inflammatory disease. Th1 polarization of the immune system is associated with the induction of psoriatic inflammation by activating various cells including neutrophils, macrophages, and T lymphocytes to release cytokines such as interferon (IFN)-γ, tumor necrosis factor (TNF)-α, and IL-2 [46]. These Th1 cytokines are also crucial in the formation and progression of atherosclerotic plaque which is important in the pathogenesis of atherosclerosis [51].

Recently, the cross-talk between neutrophil and neutrophil-associated proteins has been suggested to play a pivotal role in the pathogenesis of psoriasis and cardiometabolic disease. Among various molecules, S100A8/A9 has been identified to have a strong association with the severity of both psoriasis and vascular inflammation [52]. In addition, neutrophil extracellular traps (NETs) known to play important roles in antimicrobial defense could serve as sources of self-antigen associated with the development of autoimmune diseases such as psoriasis and atherosclerosis. NETs can directly lead to endothelial dysfunction and plaque rupture in human carotid plaque [53]. Moreover, the presence of NET in psoriatic lesions have been observed in a previous study [54], further potentiating a possible pathogenic association between psoriasis and atherosclerosis.

**Figure 1:**
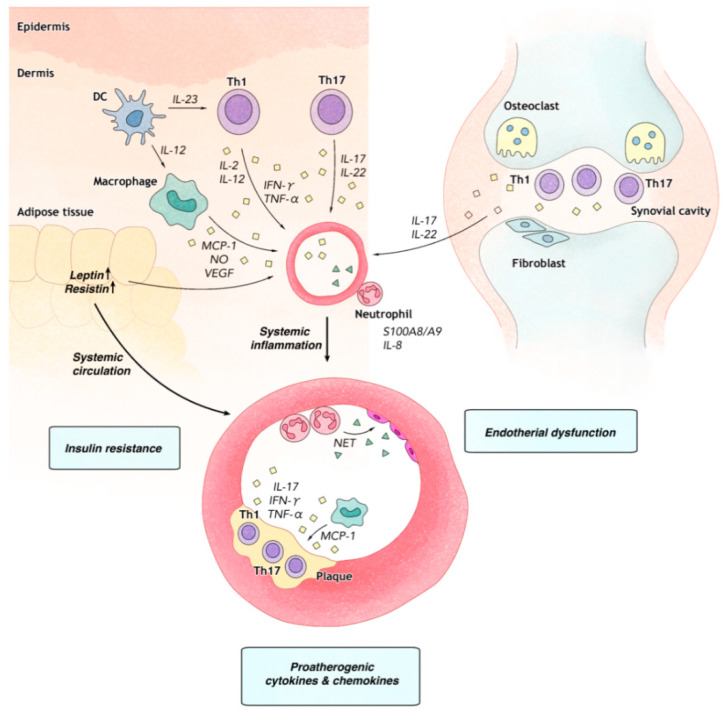
Psoriasis, psoriatic arthritis, and atherosclerosis have the common underlying pathomechanisms. In psoriatic plaque (**left**) and synovial cavity with psoriatic arthritis (**right**), myeloid dendritic cells (DC) stimulate naïve T-cells to differentiate into type 1 helper T (Th1) and type 17 helper T (Th17) cell subtypes. Th1 cells secrete tumor necrosis factor-α (TNF-α) and interferon gamma (IFN-γ), leading to keratinocyte and synovial fibroblast activation. Th17 cells secrete IL-17 and IL-22, which promote proliferation of keratinocytes and synovial fibroblasts and angiogenesis. Macrophages cooperates with Th1- and Th17-mediated inflammation by releasing monocyte chemoattractant protein (MCP-1), nitric oxide (NO), and vascular endothelial growth factor (VEGF), which further contribute to angiogenesis. Neutrophil-derived S100A8/A9 and IL-8 accelerates vascular inflammation. In atherosclerotic plaque (**bottom**), Th1 cells secrete TNF-α and IFN-γ, leading to endothelial activation and promote atherosclerosis. Leptin and resistin produced in adipose tissue enters into systemic circulation and promotes endothelial dysfunction, insulin resistance, and formation of atherosclerotic plaque. Recruitment of neutrophils on the plaque release neutrophil extracellular traps (NETs), which subsequently induces endothelial dysfunction and rupture of plaque. Macrophage-released MCP-1 synergistically promotes the formation of atherosclerotic plaque along with other pro-atherogenic cytokines in patients with psoriasis and psoriatic arthritis.

Boehncke et al. [55] have suggested a model of psoriatic march for explaining the pathogenic association between psoriasis and CVD. According to their model, systemic inflammation in psoriasis results in insulin resistance, which elicits endothelial dysfunction. Indeed, chronic inflammation in both psoriasis and atherosclerosis can induce the production of adipokines and proinflammatory cytokines, resulting in insulin resistance and further endothelial dysfunction that can drive CVD. A subtype of visceral adipose tissue can also produce a significant amount of adipokine and chemokines such as MCP-1 and IL-8 known to stimulate atherosclerosis [56]. Among various adipokines, leptin shows increased levels in sera of patients with psoriasis. Increased leptin levels are correlated with the severity of psoriasis [57]. Increased expression levels of leptin and resistin can activate the expression of proinflammatory cytokines such as MCP-1, IL-6, IL-2, and TNF-α, all of which could act as pro-atherogenic molecules and promote vascular inflammation via monocyte migration and activation of macrophage [58]. Moreover, adipokines could alter the effective functions of insulin on vessels through affect the capillary recruitment [59]. These causal links could induce metabolic syndrome or atherosclerosis that can lead to MI or stroke in patients with psoriasis and PsA (Figure 1).

## 3. The Risk of Cancer in Psoriasis and Psoriatic Arthritis

The risk of cancer in psoriasis itself is challenging to evaluate. Nonetheless, a population-based cohort study in the United Kingdom has found that patients with psoriasis have an increased risk of nonmelanoma skin cancer (NMSC) and lymphoma [60]. Of note, patients who have received systemic therapy or phototherapy are at higher risk of NMSC and lymphoma than those that are not receiving such therapy [60]. In addition, patients with moderate to severe psoriasis have higher risk of NMSC than controls [60].

A recently published meta-analysis has assessed the risk of cancer in patients with psoriasis and found that the prevalence of overall cancer is 4.78% in patients with psoriasis [61] (Table 3). In patients with psoriasis, the RR of overall cancer was 1.21 (95% CI: 1.11–1.33). It was decreased to 1.14 (95% CI: 1.04–1.25) when keratinocyte cancer was excluded. Specifically, increased risks of keratinocyte cancer (RR: 2.28; 95% CI: 1.73–3.01), lymphoma overall (RR: 1.56; 95% CI: 1.37–1.78), lung cancer (RR: 1.26; 95% CI: 1.13–1.40), and bladder cancer (RR: 1.12; 95% CI: 1.04–1.19) were observed in patients with psoriasis [61]. A recent meta-analysis by Wang et al. [62] on psoriasis according to disease severity has found that the risk of NMSC in patients with moderate to severe psoriasis (RR: 1.82; 95% CI: 1.38–2.41) is higher than that in patients with mild psoriasis (RR: 1.61; 95% CI: 1.25–2.09). In those with NMSC, the risk of squamous cell carcinoma (RR: 2.08; 95% CI: 1.53–2.83) was significantly higher than the risk of basal cell carcinoma (RR: 1.28; 95% CI: 0.81–2.00) [62]. Collectively, these results suggest that patients with psoriasis have an increased risk of cancer, especially keratinocyte cancer and lymphoma. Although the risk of solid organ cancer in patients with psoriasis was slightly increased, slightly different RRs of each cancer was observed in various studies [60,61,63].

The studies on the risk of cancer in patients with PsA are relatively few when compared to that of psoriasis. The risk of keratinocyte cancer is also significantly increased in patients with PsA (RR: 1.22; 95% CI: 0.89–1.66) [54]. Egeberg et al. [64] have also found the adjusted IRR of melanoma in patients with PsA (IRR: 1.36, 95% CI: 0.94–1.99) is higher than that in patients with mild psoriasis (IRR: 1.19; 95% CI: 1.03–1.37) or severe psoriasis (IRR: 1.09; 95% CI: 0.75–1.58). Although the risk of overall cancer in PsA was not increased, a recent meta-analysis by Vaengebjerg et al. [61] observed that patients with PsA are associated with an increased risk of breast cancer (RR: 1.73; 95% CI: 1.15–2.59).

The increased risk of cancers in psoriasis and PsA could be generally explained by the chronic inflammatory nature of psoriasis itself and the requirement for long-term therapy with immunosuppressive agents and/or phototherapy. We also observed an increased risk of cancer in patients with more severe form of psoriasis, including patients with severe psoriasis and PsA who required more long-term use and high-cumulative dose of immunosuppressants. Findings of this review suggest that the use of immunosuppressant might affect the development of cancer in patients with psoriasis.

Various studies have suggested that the risk of NMSC, especially squamous cell carcinoma, in patients with psoriasis is associated with ultraviolet irradiation during phototherapy, especially psoralen and ultraviolet A (PUVA). While the treatment with narrow band UVB has not been associated with an increased risk of skin cancer [65], PUVA treatment could trigger p53 mutation that can lead to the potential development of NMSC in patients with psoriasis. Indeed, patients receiving PUVA therapy have been found to have an increased risk of NMSC, especially of SCC [66,67]. In addition, patients frequently visiting dermatologists due to their psoriasis might lead to a higher detection rate of NMSC in this group [61]. Moreover, decreased expression of filaggrin in the lesional skin of psoriasis due to secreted cytokines including IL-4, IL-13, IL-17, and TNF-α might increase the risk of skin cancer by increasing UV sensitivity of patients with psoriasis [64].

The possible pathomechanism for increased risk of lymphoma in psoriasis might be explained by antigenic stimulation, a hypothesis proposing that prolonged immune-stimulating conditions might increase the risk of malignancy [68]. In fact, various studies have suggested that persistent immune stimulation could induce the development of a dominant clone that leads to lymphomagenesis [69,70]. Moreover, prolonged use of systemic immunomodulatory medications such as methotrexate and cyclosporine might be another possible cause for the increased risk of cancer in patients with psoriasis [71,72]. A systematic review by Peleva et al. [73] has revealed that patients treated with TNF inhibitors show a greater risk of NMSC, especially squamous cell carcinoma, than the general population in the United States. However, the majority of the reported literature has consistently suggested that treatment with biological therapies is not associated with an increased risk of cancer in patients with psoriasis and PsA [61,74,75].

Overall, it is challenging to exactly evaluate the precise effect of psoriasis and PsA on tumorigenesis. When interpreting the result of cancer risk in patients with psoriasis and PsA, diverse study designs and potential roles of common risk factors of cancer including smoking, alcohol consumption, and previous medications should be considered. Further studies are needed to in the future clarify this association by adjusting for confounding factors such as smoking, alcohol consumption, and obesity in cancer development.

## 4. The Risk of Infection in Psoriasis and Psoriatic Arthritis

Patients with psoriasis are vulnerable to systemic infections (Table 4). The risk of acute infection in psoriasis is twice increased in patients with psoriasis than in the control population [77]. Moreover, the incidence of serious infection is higher in patients with psoriasis than in the control population [78]. An increased risk of serious infection was observed in overall patients with psoriasis (hazard ratio (HR): 1.21; 95% CI: 1.18–1.23), in those with mild psoriasis (HR: 1.18; 95% CI: 1.16–1.21), and in those with moderate to severe psoriasis (HR: 1.63; 95% CI: 1.52–1.75) [78]. The underlying mechanism for the elevated infection risk in patients with psoriasis could be explained by their immune dysregulation. Increased levels of TNF-α and IL-6 before the onset of infection are associated with an increased risk of pneumonia requiring hospitalization [79], suggesting that overproduction of these cytokines can trigger bacterial invasion and the development of pneumonia in patients with psoriasis.

Concerning viral hepatitis, only a few studies have reported the prevalence of viral hepatitis in patients with psoriasis [80,81]. Yang et al. [81] have both hepatitis B virus (HBV) and hepatitis C virus (HCV) infections in Taiwanese patients with psoriasis show increased prevalence. However, Cohen et al. [80] have only found an association between HCV infection and psoriasis, consistent with previous studies [82,83]. In that study by Cohen et al. [80], the prevalence of HCV infection was 1.03% in those with psoriasis and 0.05% in the control population. The association between HCV infection and psoriasis remained significant after adjusting for IFN-α therapy and smoking [80]. Imafuku et al. [84] found that HCV antibody-positive patients have a twice higher risk of having psoriasis than HCV negative controls. This association did not change even after excluding patients with psoriasis after initiation of IFN therapy. Moreover, HCV positive ratio in patients with psoriasis showed an increasing quadratic curve with age, implying that HCV infection affected de novo onset of psoriasis, especially late-onset psoriasis in HCV positive patients [84]. The possible role of HCV infection in developing psoriasis has been studied by Chun et al. [85]. They found that the psoriatic skin of HCV-positive patients showed increased mRNA expression levels of cathelicidin, toll-like receptor 9, and IFNγ than that psoriatic skin of HCV-negative patients, suggesting that activation of inflammatory cytokines in HCV infected patients could enhance their susceptibility to psoriasis [85].

In patients with psoriasis, the risk of infection could be increased by the use of diverse immunosuppressive anti-psoriatic therapies. Indeed, all patients receiving immunosuppressive therapy are recommended to screen for HBV infection, HCV infection, and tuberculosis before the therapy [86]. Systemic immunosuppressive medications Tsuch as cyclosporine and methotrexate could lead to reactivation of HBV [87]. Cho et al. [88] reported that among seven patients with psoriasis treated with TNF inhibitor, three patients experienced the reactivation of HBV in their case series. However, most studies have reported that the risk of reactivation of HBV seems to be relatively low in patients with psoriasis treated with TNF inhibitor [89,90]. Low screening rate for HBV infection before therapies could reactivate HBV infection, which could result in potential complications such as liver cirrhosis, hepatic failure, and fulminant hepatitis [87].

TNF, a key inflammatory cytokine, plays a pivotal role in the protection against Mycobacterium tuberculosis infection by prohibiting dissemination of bacteria and granuloma formation [91,92]. As TNF inhibitors such as infliximab, etanercept, and adalimumab have been widely used in the management of psoriasis, the association between TNF inhibitors and the risk of tuberculosis has been analyzed. One study by the Spanish Society of Rheumatology Database on Biologic Products (BIOBADASER) estimated that the possible incidence of TB associated with infliximab use was 1893 cases of tuberculosis per 100,000 patient-years in the year 2000 [93]. After that study was published, more careful screening and selection with regard to tuberculosis have been conducted in clinical settings, resulting in decreased incidence of tuberculosis in patients treated with biologic therapy.

Among TNF inhibitors, etanercept is associated with the lowest incidence of tuberculosis and a longer duration of lag time for reactivation of tuberculosis [94,95]. The recently published study conducted in South Korea found that ustekinumab, an IL-12/23 inhibitor, did not increase the risk of tuberculosis when compared that with the general population based on the real-world data [96]. Fowler et al. [97] also found that the newer class of biologics in psoriasis, IL-17 inhibitors, did not increase the risk of tuberculosis for psoriasis. Furthermore, they suggested that when there is the proper treatment for latent tuberculosis infection, treatment with IL-17 inhibitors could be safely conducted in psoriasis patients with latent tuberculosis infection [97]. Yet, the possible risk of infection when using biologics has resulted in clinicians to routinely screening for HBV, HCV, and tuberculosis.

As novel biologics are widely used as time goes by, a recent study from the British Association of Dermatologists Biologic Interventions Register (BADBIR) has analyzed the risk of serious infection in psoriasis patients treated with biologics [98]. In that study, the risk of serious infection was not significantly increased in patients receiving etanercept (HR:1.10; 95% CI: 0.75–1.60), adalimumab (HR: 0.93; 95% CI: 0.69–1.26), or ustekinumab (HR: 0.92; 95% CI: 0.60–1.41) than in those receiving non-biologic systemic agents [98]. In contrast, results from Psoriasis Longitudinal Assessment and Registry (PSOLAR) have suggested an increased risk of serious infection in patients treated with infliximab and adalimumab than in patients receiving non-biological and nonmethotrexate therapies [99]. In addition, treatment with ustekinumab or etanercept was not associated with an increased risk of serious infection [99]. The most recent epidemiological study by Li et al. [100] has compared RRs of serious infections in those receiving various biologics. It was found that IL-12/23 inhibitors were associated with lower risks of serious infection in biologic-naïve patients with psoriasis and PsA than TNF inhibitors and IL-17 inhibitors [100]. However, when risks of serious infection were compared among biologic-experienced patients, there was no difference in the risk of serious infection among IL-17, IL-12/23, and TNF inhibitors [100]. As the new biologics have been developed, questions about whether these newly developing agents could increase the risk of infection in psoriasis should be further evaluated using real-world clinical dataset.

In contrast to research on infection in patients with psoriasis, only a few studies have been conducted to identify the risk of infection in patients with PsA. Eder et al. [101] reported that patients with PsA showed an increased risk of infection that required antibiotics (OR: 1.7; 95% CI: 1.00–2.77). As patients with PsA tend to receive more biologics than patients with psoriasis, they are at an increased risk of developing adverse events including infections [102]. The study by Haddad et al. [103] also found that patients with PsA showed an increased incidence of infection than patients with psoriasis. Among patients with PsA, patients receiving biologics showed the increased HR of infection (HR: 1.56; 95% CI: 1.22–2.0) when compared with patients receiving non-biologics [103]. Therefore, the physician should always cautiously monitor for the occurrence of any infection when using biologics in PsA. With regards to HCV in PsA, a study by Taglione et al. [104] observed the increased prevalence of HCV infection in patients with PsA than patients with rheumatoid arthritis and with healthy controls. However, the study by Palazzi et al. [105] did not find a significant difference in the prevalence of HCV infection between patients with PsA and healthy control. As results from previous studies are somewhat contradictory, further epidemiological studies to clarify this association is needed in the future.

## 5. The Risk of Autoimmune Diseases in Psoriasis and Psoriatic Arthritis

Patients with psoriasis have been reported to have a higher frequency of having autoimmune diseases than healthy controls [108,109]. In a Danish population, children and adolescents with psoriasis showed increased risks of having autoimmune disorders, including rheumatoid arthritis (OR: 6.61; 95% CI: 2.75–15.87) and vitiligo (OR: 4.76; 95% CI: 1.71–13.20), than healthy controls [110]. Table 5 summarizes the recently reported studies identifying the risk of autoimmune disorders in patients with psoriasis and PsA.

With regard to autoimmune bullous disease (AIBD), the first case report of bullous eruption in psoriasis was reported in 1929 [119]. Since then, various studies have been conducted to identify the possible association between AIBD and psoriasis. The study by Ohata et al. [120] have reported that among 145 patients with coexisting AIBD, 63.4% of patients have bullous pemphigoid (BP) and 37.2% of patients have anti-concomitant laminin γ1 pemphigoid concomitantly. One study has analyzed comorbid diseases in patients with BP and found that the risk of psoriasis is significantly increased in patients with BP (OR: 2.02; 95% CI: 1.54–2.66) than in healthy controls [121]. In a case-control study of 287 patients newly diagnosed with BP and 1373 matched controls, the prevalence rate of psoriasis was increased in patients with psoriasis than that in controls (OR: 4.4; 95% CI: 2.2–8.9) [122]. In particular, patients with both BP and psoriasis were significantly younger than patients that had only BP [122]. In that study, authors suggested that altered antigenicity of basement membrane in patients with psoriasis at a younger age might have induced the activation of antibasement membrane antibody, resulting in the development of BP at a younger age [122]. A recent systematic review and meta-analysis have shown that the prevalence of psoriasis in BP is increased compared to that in controls (OR: 2.5; 95% CI: 1.4–4.6) [113]. In particular, the risk of predisposition in males was statistically higher than that in females (OR: 1.73; 95% CI: 1.1–2.7) [113]. Although further studies are warranted in the future to determine whether psoriasis might induce the development of BP or vice versa, the possible underlying mechanism of action between autoimmune bullous disease and psoriasis can be explained by autoantibodies, cytokines, and drugs. Destruction of laminin at the basement membrane of psoriatic skin lesions has been observed [123]. McFadden et al. [124] have suggested that disruption of laminin might result in instability and proliferation of keratinocytes, leading to the development of psoriasis. Damage to the basement membrane in psoriasis might induce the development of diverse anti-basement membrane zone antibodies that might be associated with the induction of AIBD. In addition, IL-1 functions as a key cytokine in both psoriasis and bullous disease. Yano et al. [125] have postulated that IL-1 is associated with the initiation and development of psoriatic lesion as IL-1 regulated genes are involved in proteolysis, adhesion, signal transduction, proliferation, and epidermal differentiation. Moreover, increased expression of IL-1β has been observed in lesional blister fluid than in serum of patients with BP [126]. Based on these findings, IL-1 might exert a role in the development of these two disorders. In addition, as patients with psoriasis receive diverse treatment options including systemic immunosuppressants and phototherapies, these treatment options might trigger the development of AIBD. Indeed, UV irradiation including ultraviolet B and PUVA might alter antigenicity of the basement membrane, thus affecting the development of bullous disease [107,127].

Vitiligo, another autoimmune cutaneous disorder, is associated with psoriasis. A nationwide study has shown an increased risk of vitiligo (OR: 5.94; 95% CI: 3.79–9.31) in patients with psoriasis [108]. A recent study by Yen et al. [115] has also found a significantly increased risk of vitiligo in patients with psoriasis (OR: 2.29; 95% CI: 1.56–3.37) and an increased risk of psoriasis in patients with vitiligo (OR: 3.43; 95% CI: 1.86–6.33), suggesting that these two disorders are associated with each other. Shared genetic loci including rs9468925 and HLA-C/HLA-B have been observed in psoriasis and vitiligo [128]. Cell-mediated immune pathways including Th1 and Th17 pathways shared by these two disorders might also explain their association. Treatment with IFN-α, an important cytokine in the pathogenesis of both vitiligo and psoriasis, for HCV and HBV infection can induce both psoriasis and vitiligo [129,130,131]. Moreover, narrow band ultraviolet B phototherapy and Janus kinase inhibitor can be used for treating both psoriasis and vitiligo [132,133,134].

In addition, psoriasis and inflammatory bowel disorders (IBD) including Crohn’s disease (CD) and ulcerative colitis (UC), share genetic and inflammatory mechanisms. Although a nationwide study in Taiwan did not observe a significant association between CD and psoriasis, other studies reported a higher risk of having CD in patients with psoriasis [135,136]. Among IBD, psoriasis might be more strongly associated with CD (OR: 2.49; 95% CI: 1.71–3.62) than UC (OR: 1.64; 95% CI: 1.15–2.23) [136]. Altered Th1 and Th17 immune pathways are not only involved in the pathogenesis of psoriasis, but also involved in the pathogenesis of CD. Among various cytokines involved in these pathways, TNF-α and IL-23 are crucial cytokines involved in psoriatic skin inflammation and intestinal inflammation. TNF-α inhibitors show beneficial effects both on psoriasis and IBD. Various studies have reported a single-nucleotide polymorphism in IL23R both in psoriasis and IBD [137,138,139]. In addition, treatment with IL-17 inhibitors in psoriasis and PsA can induce the development or aggravation of IBD in susceptible patients, although whether it is the development of IBD observed in predisposing patients or inhibiting IL-17 that causes the de novo development of IBD needs to be further determined in future studies [140]. Patients with psoriasis are also likely to have alopecia areata, an autoimmune hair loss condition. A nationwide population-based study has found that the risk of alopecia areata in patients with psoriasis is increased than in a control population (OR: 4.71; 95% CI: 2.98–7.45) [108]. In accordance with this study, Wu et al. [109] also found an increased risk of alopecia areata in patients with psoriasis (OR, 2.4; 95% CI, 1.9–2.9).

Vassilatou et al. [141] enrolled 114 patients with psoriasis and 286 body mass index- and age-matched control subjects and found that there was no statistically significant difference in the prevalence of autoimmune thyroiditis between patients with psoriasis (20.2%) and control subjects (19.6%), in accordance with Gul et al. [142], who reported no difference in the positivity of antithyroid peroxidase antibody or antithyroglobulin antibody between patients with psoriasis and controls. However, a retrospective cross-sectional study with a total of 856,615 participants, including 9654 patients with psoriasis and 1745 patients with Hashimoto’s thyroiditis, reported that patients with psoriasis show an increased risk of Hashimoto’s thyroiditis than controls even after adjusting for gender, age, psoriatic arthropathy, and the use of anti-psoriatic systemic medications (OR: 2.49; 95% CI: 1.79–3.48) [143]. Autoimmune thyroid disease and psoriasis are both characterized by alteration in Th1-medicated immune pathways. Indeed, Th1 predominance affects the initiation and development of both autoimmune thyroiditis and psoriasis under environmental triggers and genetic predisposition. As anti-psoriatic biologic agents have the potential to reduce the systemic inflammation of psoriasis, we can suppose that using biologics could decrease the development of various autoimmune disorders. However, to date, there are only some case reports regarding the development of thyroiditis after using biologics in psoriasis [144,145]. However, the study by Kiguradze et al. [143] reported that there was no significant association between the use of systemic anti-psoriatic biologics and the development of Hashimoto’s thyroiditis. Further long-term epidemiological studies assessing the possible pathogenic links between the development of psoriasis and autoimmune thyroiditis are needed in the future.

In particular, patients with PsA showed increased risks of having autoimmune diseases than patients with psoriasis [109]. Wu et al. [109] reported that patients with PsA showed an increased risk for development of various autoimmune disorders including rheumatoid arthritis, celiac disease, systemic sclerosis, Crohn’s disease, Sjogren syndrome, ulcerative colitis, systemic lupus erythematosus, Addison disease, giant cell arteritis, pulmonary fibrosis, Hashimoto thyroiditis, chronic glomerulonephritis, graves disease, type 1 diabetes mellitus, immune thrombocytopenic purpura, multiple sclerosis, and hemolytic anemia when compared to patients with psoriasis. As patients with PsA have relatively higher levels of systemic inflammation than patients with psoriasis only [109], this could be a possible explanation for this association. With regards to IBD, a meta-analysis confirmed the increased odds of IBD in patients with PsA [116]. Although there are some conflicting results regarding the association between autoimmune thyroiditis and psoriasis, the relevant association between autoimmune thyroiditis and PsA has been consistently found in various studies. Antonelli et al. [146] have reported that the prevalence of thyroid autoimmunities such as positive antithyroid peroxidase antibody and hypoechoic thyroid pattern is increased in male and female PsA patients. In addition, subclinical hypothyroidism was more frequently observed in female PsA patients than in the general population [146]. A recent longitudinal prospective study has found that patients with PsA show increased rates of hypothyroidism, thyroid dysfunction, positive antithyroid peroxidase, and small hypoechoic thyroid pattern than control subjects [147]. In particular, such increases compared to control subjects were frequently observed in females than in males [147]. Among various cytokines and chemokines related to the Th1 pathway, serum levels of C-X-C motif chemokine ligand 10, a prototype chemokine of the Th1 pathway, are increased in patients with PsA than in control subjects [148]. PsA patients with autoimmune thyroiditis showed higher CXCL10 levels than patients with PsA [148].

## 6. The Risk of Psychiatric Diseases in Psoriasis and Psoriatic Arthritis

Due to the chronic nature of the disease itself, patients with psoriasis suffer from significantly poor quality of life regardless of the extent of involved body surface area [149]. Increased psychosocial burden associated with psoriasis has resulted in many researchers to analyze the risk of psychiatric comorbid disease in psoriasis. Table 6 summarizes nationwide population-based cohort studies analyzing the risk of each psychiatric disorder in patients with psoriasis and PsA.

The prevalence of depression in patients with psoriasis was higher than that in the control population, ranging from 2.10% to 33.7% according to measures for depression and study populations [108,150,151]. Moreover, patients with psoriasis show an increased prevalence of depression than patients with other dermatological disorders including nonmelanoma skin cancer, eczema, acne, and atopic dermatitis [152,153]. A systematic review and meta-analysis have been performed to determine the prevalence and odds of depressive symptoms and clinical depression, revealing that patients with psoriasis show more depressive symptoms than control populations (standardized mean difference: 1.16; 95% CI: 0.67–1.66) [154]. Patients with psoriasis are more likely to experience depression (OR: 1.57; 95% CI: 1.40–1.76) [154]. They are also more likely to be treated with antidepressants (OR: 4.24; 95% CI: 1.53–11.76) than the control population [154]. It has been reported that over 10% of patients with psoriasis experience clinical depression [154].

Compared to studies on depression, fewer studies have reported the prevalence of anxiety disorders in psoriasis. Nonetheless, there has been an internal consistency with regard to an increased prevalence of anxiety disorders in patients with psoriasis compared to controls. A nationwide population-based cohort study has revealed that the risk of anxiety disorders is increased in patients with psoriasis than in controls [155].

In patients with schizophrenia, a nationwide retrospective cohort study has reported that the HR of psoriasis is 2.35 (95% CI: 1.83–3.01) [163]. In addition, the prevalence of schizophrenia in patients with psoriasis is higher than that in a control population [161]. Moreover, patients with psoriasis show an increased risk of schizophrenia than controls [161]. Especially, psoriasis patients having comorbid diseases such as cerebrovascular disease (OR: 2.01; 95% CI: 1.11–3.65) and chronic pulmonary disease (OR: 1.64; 95% CI: 1.07–2.49) show an increased risk for schizophrenia [161].

With regard to suicidal ideation, the percent of suicidal ideation is higher in patients with a history of psoriasis (4.9%) than in patients with no history of psoriasis (3.7%), although the difference is not significant [157]. A meta-analysis conducted by Chi et al. [164] has suggested the risk of suicide and suicide attempt or suicidality in patients with psoriasis is not increased. On the contrary, another meta-analysis analyzing the association between psoriasis and suicidality has shown that risks for suicidal ideation (OR: 2.05, 95% CI: 1.54–2.74) and suicidal behaviors (OR: 1.26; 95% CI: 1.13–1.40) are increased in patients with psoriasis compared to controls [165]. In addition, patients with psoriasis have higher risks of attempt suicide (pooled OR: 1.32; 95% CI: 1.14–1.54) and complete suicide (pooled OR: 1.20; 95% CI: 1.04–1.39) than the control population [165]. Considering these inconsistent results from available literature, further studies are warranted to clearly identify this association. Nonetheless, if left untreated, depression and anxiety could result in suicidality. Thus, these mental health disorders should be critically monitored throughout the treatment for psoriasis.

Psychiatric disorders are also commonly observed in patients with PsA. The estimated prevalence of at least mild depression in patients with PsA was estimated as 20% (95% CI: 8–35%) in a meta-analysis [166]. The presence of PsA in patients with psoriasis can also increase the risk of depression. A population-based cohort study found that relative risk for depression was increased as 1.22 (95% CI: 1.16–1.29) in patients with PsA [159]. In addition to depression, the pooled prevalence of at least moderate anxiety was estimated as 21% (95% CI: 14%–29%) in patients with PsA [166].

**Figure 2:**
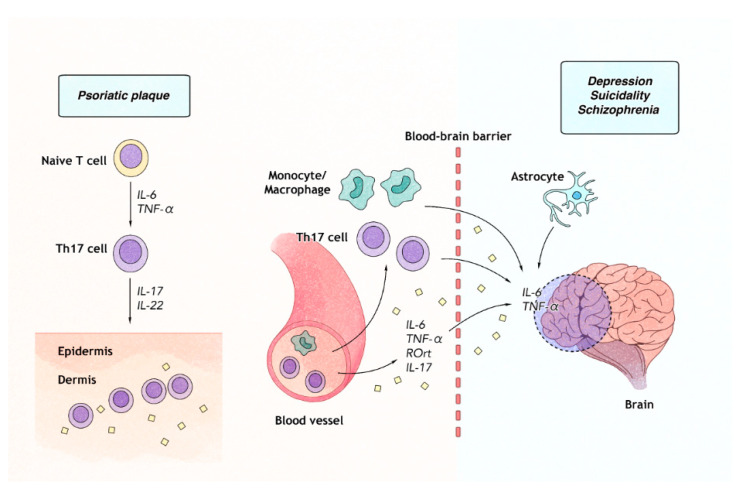
IL-6 and TNF-α-mediated inflammation: a possible link between psoriasis and accompanying psychiatric disorders. In psoriatic plaque (**left**), naïve T-cells to differentiate into type 17 helper T (Th17) cells under IL-6 and TNF-α-prone microenvironment. IL-6 and TNF-α stimulates Th17 cells proliferation and potentiates IL-17 mediated inflammation in psoriatic lesions. In systemic circulation *(***middle**)*,* elevated serum levels of IL-6, and TNF-α are commonly observed in psychiatric disorders such major depressive disorders. IL-6, and TNF-α are increased in the brain tissue (**right**) of patients who attempted suicide. The increased levels of serum IL-6, TNF-α, retinoic acid receptor-related orphan receptor gamma t (ROγt) and IL-17 are observed in patients with major depressive disorders, and these molecules appear to contribute to the development of both psoriasis and psychiatric comorbidities by passing through blood-brain barrier and influencing each other.

Psoriatic proinflammatory cytokines such as IL-6, IL-17, and TNF-α are also associated with various psychiatric conditions (Figure 2). Chen et al. [167] have found that patients with depression are more likely to have an increased number of peripheral Th17 cells by flow cytometric analysis. In addition, levels of serum retinoic acid receptor-related orphan receptor gamma t and IL-17 were elevated in patients with major depressive disorder, suggesting a role of Th17 cells in the pathogenesis of major depressive disorder [168]. Moreover, psoriasis mice model has shown that the expression of IL-17A in peripheral immune cells is increased [168]. Such an increase is associated with inflammatory mediators in different brain regions and depression-like symptoms, implying a potential link between psoriatic inflammation and depression-like symptoms in mice [168]. IL-6, another pro-inflammatory cytokine, is involved in the maturation of naïve T cells to Th17 cells. It is associated with both suicidality and psoriasis. The level of IL-6 is elevated in the central nervous system of subjects who have attempted suicide than in controls [169]. Moreover, mRNA and protein expression levels of IL-6 and TNF-α are increased in the Brodmann area 10 of suicide teenage victims than in healthy controls [170]. Serum levels of IL-6 and TNF-α are increased in active psoriatic patients than in controls [171]. With regard to schizophrenia, the Th17 signaling pathway, IL-6, and TNF-α are also associated with the development and aggravation of schizophrenia [172,173]. These findings suggest that inflammatory mediators including IL-17A, IL-6, and TNF-α might exert a crucial role in the possible link between psoriasis and these psychiatric disorders.

Recent studies evaluating the treatment efficacy of newly developed biologics and oral small molecule inhibitors have observed that these agents can improve anxiety and depression scales in addition to clinical improvement. Indeed, treatment with brodalumab, a monoclonal antibody against IL-17 receptor A, has resulted in significant decreases in mean scores of hospital anxiety and depression scale than placebo [174]. Treatment with apremilast, a PDE4 inhibitor, has also resulted in a significant improvement in patient-reported health-related quality of life at week 16 than placebo [175]. This improvement was maintained through week 32 [175]. Treatment with ustekinumab, a fully human monoclonal antibody against the p40 subunit, which binds to IL-12 and IL-23, has resulted in a significant improvement in symptoms of depression and anxiety in moderate-to-severe patients with psoriasis [176]. Based on these findings, it is anticipated that the prevalence of mood disorder might be decreased due to the development of these new anti-psoriatic agents. However, depression after the use of apremilast has been reported [177]. Thus, precaution is required when using this agent in susceptible patients. In addition, mood change can also occur after the use of acitretin. Thus, patients with psoriasis treated with acitretin should be carefully monitored for mood instability [86].

Patients with psoriasis have an increased risk of sleep disorder [108]. Moreover, sleep apnea characterized by a chronic intermittent collapse of upper respiratory airways during sleep is also associated with systemic inflammation and psoriasis. In addition, the IRR was 1.62 in those with mild psoriasis, and 2.04 in those with severe psoriasis, compared to patients with sleep apnea without continuous positive airway pressure (CPAP) therapy [162]. Moreover, patients with sleep apnea receiving CPAP therapy who were regarded as having more severe sleep apnea showed increased risks of having mild psoriasis (IRR: 1.82; 95% CI: 1.43–2.33) and severe psoriasis (IRR: 3.27; 95% CI: 2.03–5.27) [162]. A recent systematic review has reported increased prevalence of obstructive sleep apnea (prevalence rate in psoriasis 36.0%–81.8% versus general population 2%–4%) and restless legs syndrome (prevalence rate in psoriasis 15.1%–18% versus 5%–10% in the general population) in patients with psoriasis, implying possible links of psoriasis and with obstructive sleep apnea and restless leg syndrome [178].

In patients with PsA, the incidence rate ratio (IRR) for sleep apnea was 1.30 when compared to controls [162]. The IRR for sleep apnea in PsA was higher than that of psoriasis [162]. In addition, IRR was 1.94 in those with PsA compared to patients with sleep apnea without CPAP therapy [162]. Patients with sleep apnea receiving CPAP therapy also showed increased risks of having PsA (IRR: 5.59; 95% CI: 3.74–8.37) [162]. However, psoriasis and PsA showed no significant association with other sleep disorders such as insomnia, periodic limb movement disorder, narcolepsy, or shift work disorder in that systematic review [178]. Possible pathomechanisms between psoriasis and PsA, and obstructive sleep apnea can be explained by inflammatory cytokines. Intermittent hypoxia and oxidative stress in patients with obstructive sleep apnea can result in the activation of nuclear factor-κB, which could further induce downstream release of inflammatory cytokines such as TNF-α and IL-6. These cytokines are well-known to be associated with the pathogenesis of psoriasis and PsA [179,180,181]. Although possible pathomechanisms between these disorders remain to be further identified, the relationship might be explained by their shared pathogenic inflammatory pathways.

## 7. Conclusions

Nowadays, psoriasis is considered a chronic-recurrent systemic inflammatory disorder. The chronic inflammatory nature of psoriasis itself affects functions of multiple organs in the body beyond the skin. In this review, we also found clear links of psoriasis and PsA with various comorbid disorders, including CVD, metabolic disease, cancers, infections, autoimmune disease, and psychiatric diseases. Patients with more severe psoriasis and PsA and longer disease duration tend to have an increased risk of comorbidities. Moreover, psoriasis patients with comorbidities, especially those with cerebrocardiovascular comorbidities, showed even higher mortality and hospitalization risks.

A large number of exploration studies have revealed pathomechanisms of psoriasis and concluded that psoriasis is the result of multiple interactions among genetic predisposition, environmental triggering factors, and immune alteration. In addition to T cells, diverse effector cells and related inflammatory molecules are involved in the initiation and development of psoriasis. The increased risk of various comorbidities in patients with psoriasis and PsA might be explained by the fact that diverse cellular and molecular pathogenic pathways are shared by those with several comorbid disorders and those with psoriasis and PsA. Persistent chronic inflammation in psoriasis and PsA can also eventually lead to systemic inflammation, which results in the development of diverse comorbidities. The use of systemic anti-psoriatic drugs and phototherapy is also associated with increased risks of some comorbidities including infection and cancer. On the other hand, novel anti-psoriatic biologics can decrease the risk of comorbidities, including CVD, metabolic diseases, and psychiatric disease in patients with psoriasis by decreasing the degree of systemic inflammation. However, further research is needed to draw a clear conclusion concerning the treatment efficacy of newly developed biologics for psoriasis comorbidities. As patients with psoriasis and PsA are generally treated with various treatment modalities throughout their lifespan due to the chronic nature of the disease itself, additional research is needed based on real-world clinical dataset to determine whether differences in risk of comorbidities are caused only by the treatment itself or by the accumulation of multiple drugs effects.

Given that risks of comorbidities are increased in patients with psoriasis and PsA, physicians should always consider psoriasis and PsA as a multisystemic disorder and provide a generalized multidisciplinary approach to treat their possible comorbidities.

## Figures and Tables

**Table 1:** Summary of results from systematic reviews and meta-analyses evaluating the risk of cardiovascular disease in patients with psoriasis and psoriatic arthritis.

Study Number	Study Population	Identified Risk Among CV Conditions	The Relative Risk of Measures
Psoriasis			
1 [14]	Psoriasis patients: 503,686 Controls: 29,686,694	CVD, IHD, cerebrovascular disease, and CV mortality	CVD in total OR 1.4 (1.2–1.7) IHD OR 1.5 (1.2–1.9) Cerebrovascular disease OR 1.1 (0.9–1.3) CV mortality OR 0.9 (0.4–2.2)
2 [15]	Psoriasis patients + controls: 6,230,774	Stroke, MI, CVD, and CV mortality	Stroke RR 1.26 (1.12–1.41) MI RR 1.32 (1.13–1.55) CVD RR 1.47 (1.30–1.60) CV mortality RR 1.33 (1.00–1.77)
3 [16]	Psoriasis patients: 324,650 Controls: 5,309,087	MI, CAD, and stroke	Cohort studies: MI OR 1.25 (1.03–1.52), CAD OR 1.20 (1.13–1.27), stroke OR 1.02 (0.92–1.14) Cross-sectional studies: MI OR 1.57 (1.08–2.27), CAD OR 1.84 (1.09 –3.09), stroke OR 1.14 (1.08–1.19)
4 [17]	Psoriasis patients: 367,358 Controls: 9,199,656	CV events: MI, IHD, cerebral ischemic stroke, and sudden cardiac death	CV events OR 1.28 (1.18–1.38)
5 [18]	Psoriasis patients: 488,315 (mild: 327,418; severe: 12,854) Controls: 10,024,815	CVD mortality, MI, and Stroke	All psoriasis: MI HR 1.40 (1.03–1.89) and stroke HR 1.13 (1.01–1.26) Mild psoriasis: CVD mortality SMR 1.03 (0.86–1.25), MI HR 1.34 (1.07– 1.68), and stroke HR 1.15 (0.98–1.35) Severe psoriasis: CVD mortality SMR 1.37 (1.17–1.60), CVD mortality HR 1.57 (1.26–1.96), MI HR 3.04 (0.65–14.35) and stroke HR 1.59 (1.34– 1.89)
6 [19]	Psoriasis patients: 218,654 (mild: 201,239; severe: 17,415) Controls: 9,914,799	MACE: CV mortality, MI, and stroke	Mild Psoriasis: MI RR 1.29 (1.02–1.63), stroke RR 1.12 (1.08–1.16) Severe Psoriasis: MI RR 1.70 (1.32–2.18), stroke RR 1.56 (1.32–1.84), CV mortality RR 1.39 (1.11–1.74)
7 [20]	Psoriasis patients: 1,862,297 Controls: 43,407,300	MI, CVD, and CV death	Overall CV RR 1.24 (1.18–1.31), MI RR 1.24 (1.11–1.39) Vascular disease RR 1.27 (1.12–1.43), CV Mortality RR 1.41 (0.97–2.04)
8 [21]	Psoriasis patients: 326,598 Controls: 5,230,048	MI, and stroke	MI and stroke RR 1.20 (1.10–1.31) MI RR 1.22 (1.05–1.42) Stroke RR 1.21 (1.04–1.40)
**Psoriatic arthritis**			
1 [23]	Psoriatic arthritis patients: 32,973 Controls: 4,568,723	CVD, CV events Morbidity risks for MI, cerebrovascular events, and heart failure	CVD OR 1.43 (1.24–1.66) CV events 1.55 (1.22–1.96) Morbidity risk for MI OR 1.68 (1.31–2.15) Morbidity risk for cerebrovascular events OR 1.22 (1.05–1.41) Morbidity risk for heart failure OR 1.32 (1.11–1.57)

Abbreviations: CAD, coronary artery disease; CV, cardiovascular; HR, hazard ratio; IHD, ischemic heart disease; MACE, major adverse cardiovascular events; MI, myocardial infarction; OR, odds ratio; RR, relative risk; SMR, standardized mortality ratio. Values in brackets indicate 95% confidence intervals.

**Table 2:** Recent systematic reviews and meta-analyses analyzing the risk of cardiovascular risk factors in psoriasis.

Study	Population	Identified Cardiovascular Risk Factors with Relative Risk of Measures
Psoriasis		
Armstrong (2013) [28]	Psoriasis: 309,469 Control: 2,088,197	Hypertension in all psoriasis OR 1.58 (1.42–1.76); in mild psoriasis OR 1.30 (1.15–1.47); in severe psoriasis OR 1.49 (1.20–1.86)
Duan (2020) [29]	Psoriasis: 255,132 Control: 814,631	Hypertension OR 1.43 (1.25–1.64)
Armstrong (2013) [30]	Psoriasis: 314,036 Control: 3,717,217	Diabetes OR 1.59 (1.38–1.83); in mild psoriasis pooled OR 1.53 (1.16–2.04); in severe psoriasis pooled OR 1.97 (1.48–2.62)
Coto-Segura (2013) [31]	Psoriasis: 557,697 Control: 5,186,485	Type 2 diabetes pooled OR 1.76 (1.59–1.96)
Mamizadeh (2019) [32]	Psoriasis: 922,870 Control: 12,808,071	Diabetes OR 1.69 (1.51–1.89)
Armstrong (2012) [33]	Psoriasis: 201,831 Control: 1,898,169	Obesity OR 1.66 (1.46-1.89); in mild psoriasis OR 1.46 (1.17–1.82); in severe psoriasis OR 2.23 (1.63–3.05)
Miller (2013) [14]	Psoriasis: 503,686 Control: 27,686,694	Diabetes OR 1.9 (1.5–2.5); hypertension OR 1.8 (1.6–2.0), dyslipidemia OR 1.5 (1.4–1.7); obesity OR 1.8 (1.4–2.2); metabolic syndrome OR 1.8 (1.2–2.8)
Choudhary (2020) [34]	Psoriasis: 17,672 Control: 66,407	Increased systolic blood pressure OR 2.31 (1.12–4.74); diastolic blood pressure OR 2.31 (1.58–3.38); abdominal obesity OR 1.90 (1.45–2.50); Triglycerides OR 1.80 (1.29–2.51)
Phan (2020) [35]	Pediatric psoriasis: 43,808 Control: 5,384,057	Obesity OR 2.45 (1.73–3.48); diabetes OR 2.32 (1.34–4.03); hypertension OR 2.19 (1.62–2.95); hyperlipidemia OR 2.01 (1.66–2.42); metabolic syndrome OR 1.75 (1.75–7.14)
Armstrong (2013) [36]	Psoriasis: 41,853 Control: 1,358,147	Metabolic syndrome OR 2.26 (1.70–3.01)
Rodríguez-Zúniga (2017) [37]	Psoriasis: 25,042 Control: 131,609	Metabolic syndrome pooled OR 1.42 (1.28–1.65)
Singh (2017) [38]	Psoriasis: 46,714 Control: 1,403,474	Metabolic syndrome pooled OR 2.14 (1.84–2.48)
Choudhary (2019) [39]	Psoriasis: 15,939 Control: 103,984	Metabolic syndrome OR 2.077 (1.84–2.34)
**Psoriatic arthritis**		
Coto-Segura (2013) [31]	Psoriatic arthritis: 3568 Control: 13,346	Type II diabetes mellitus OR 2.18 (1.36–3.50)

Abbreviation: OR, odds ratio. Values in brackets indicate 95% confidence intervals.

**Table 3:** Summary of the systematic review and meta-analyses identifying the risk of cancers in patients with psoriasis and psoriatic arthritis.

Number	Study	Main Outcomes			
		Overall Cancer	Solid Organ Cancer	Hematologic Cancer	Skin Cancer
Psoriasis					
1 [63]	Pouplard (2013)	Overall cancer SIR 1.16 (1.07–1.25)	Respiratory tract cancer SIR 1.52 (1.35–1.71); upper aerodigestive tract cancer SIR 3.05 (1.74–5.32); urinary tract cancer SIR 1.31 (1.11–1.55); liver cancer SIR 1.90 (1.48–2.44)	Non-Hodgkin lymphoma SIR 1.40 (1.06–1.86);	BCC SIR 2.00 (1.83–2.20)
2 [76]	Trafford (2019)	Overall cancer in all severities of psoriasis RR 1.18 (1.06–1.31); severe psoriasis RR 1.22 (1.08–1.39)	Colon cancer RR 1.18 (1.03–1.35); colorectal RR 1.34 (1.06–1.70); kidney RR 1.58 (1.11–2.24); laryngeal RR 1.79 (1.06–3.01); liver RR 1.83 (1.28–2.61); pancreatic cancer RR 1.41 (1.16–1.73)	Lymphoma RR 1.40 (1.24–1.57); non-Hodgkin lymphoma RR 1.28 (1.15–1.43)	Keratinocyte cancer RR 1.71 (1.08–2.71); SCC RR 2.15 (1.32–3.50)
3 [61]	Vaengebjerg (2020)	Overall cancer RR 1.21 (1.11–1.33); cancer excluding keratinocyte cancer RR 1.14 (1.04–1.25)	Lung cancer RR 1.26 (1.13–1.40); bladder cancer RR 1.12 (1.04-1.19)	Lymphoma overall RR 1.56 (1.37–1.78); non-Hodgkin lymphoma RR 1.48 (1.30–1.69)	Keratinocyte cancer RR 2.28 (1.73–3.01)
4 [62]	Wang (2020)	N/A	N/A	N/A	NMSC RR 1.72 (1.46–2.02); risk of NMSC in moderate to severe psoriasis RR 1.82 (1.38–2.41); risk of NMSC in mild psoriasis RR 1.61 (1.25–2.09); SCC RR 2.08 (1.53–2.83); BCC RR 1.28 (0.81–2.00)
**Psoriatic arthritis**					
1 [61]	Vaengebjerg (2020)	Overall cancer RR 1.02 (0.97–1.08)		Breast cancer RR 1.73 (1.15–2.59)	

Abbreviation: BCC, basal cell carcinoma; N/A, not applicable; NMSC, non-melanoma skin cancer; RR, relative ratio; SCC, squamous cell carcinoma; SIR, standardized incidence ratio. Values in brackets indicate 95% confidence intervals.

**Table 4:** Summary of the epidemiological studies investigating the risk of infection in patients with psoriasis and psoriatic arthritis.

Study	Country	Subjects	Outcomes
**Risk of general infections in psoriasis**			
Takeshita (2018) [78]	UK	Psoriasis: 199,700 Control: 954,315	HR of serious infection in overall patients with psoriasis 1.21 (1.18–1.23); in mild psoriasis 1.18 (1.16–1.21); in moderate to severe psoriasis 1.63 (1.52–1.75); HR of opportunistic infection in moderate to severe psoriasis 1.57 (1.06–2.34); HR of herpes zoster in mild psoriasis 1.07 (1.05–1.10); in moderate to severe psoriasis 1.17 (1.06–1.30)
Kim (2019) [77]	Korean	Psoriasis: 16,383 Control: 613,144	RR of all infections 1.89 (1.83–1.94); RR of all infections except skin and soft tissue 1.58 (1.53–1.63)
**Risk of general infections in PsA**			
Pattison (2008) [106]	UK	PsA: 98 Control: 163	OR of recurrent oral ulceration 4.20 (1.96–9.0)
Eder (2011) [101]	Canada	PsA: 159 Control: 159	OR of infection that required antibiotics 1.7 (1.00–2.77)
Haddad (2016) [103]	Canada	PsA: 695 Psoriasis: 509	HR of infection for PsA versus psoriasis 1.56 (1.18–2.06)
**Risk of viral hepatitis in psoriasis**			
Taglione (1999) [107]	Italy	Psoriasis: 50 Control: 76	Prevalence of HCV was not higher in patients with psoriasis than controls.
Cohen (2010) [80]	Israel	Psoriasis: 12,502 Control: 24,287	OR of HCV in smokers 1.93 (1.30–2.67); OR of HCV in nonsmokers 2.22 (1.63–3.04) OR of HBV 1.22 (0.93–1.60)
Tsai (2011) [108]	Taiwan	Psoriasis: 51,800 Control: 207,200	OR of HCV 2.02 (1.67–2.44 OR of HBV 1.73 (1.47–2.04)
Yang (2011) [81]	Taiwan	Psoriasis: 1,685 Control: 5,055	OR of HBV or HCV in overall psoriasis 1.34 (1.04–1.73); OR of HBV or HCV in moderate to severe psoriasis 1.39 (1.06–1.83)
Imafuku (2013) [84]	Japan	Psoriasis: 717 Control: 38,057	OR of HCV 2.42 (1.82–3.21)
**Risk of viral hepatitis in PsA**			
Palazzi (2005) [105]	Italy	PsA: 100 Control: 100	No significant difference in HCV prevalence between PsA and control (*p* = 0.68)
Taglione (1999) [107]	Italy	PsA: 50 Control: 76	Prevalence of HCV in PsA was higher than controls.

Abbreviation: HBV, hepatitis B; HCV, hepatitis C; HR, hazard ratio; OR, odds ratio; PsA, psoriatic arthritis; RR, rate ratio. Values in brackets indicate 95% confidence intervals.

**Table 5:** Summary of the systematic review and meta-analyses identifying the association of psoriasis and psoriatic arthritis with various autoimmune diseases.

Study	Subjects	Outcomes
Psoriasis		
Khan (2017) [111]	Autoimmune thyroid disease: 3,535 Control: 416,649	OR of psoriasis 1.25 (1.14–1.37)
Liu (2019) [112]	Multiple sclerosis: 25,187; Control: 227,225	HR of psoriasis 1.92 (1.32–2.80)
Phan (2019) [113]	Bullous pemphigoid: 4,035; Control: 19,215	OR of psoriasis 2.5 (1.4–4.6)
Kridin (2019) [114]	Pemphigus: 12,238; Control: 87,051,168	OR of psoriasis 3.5 (1.6–7.6)
Yen (2019) [115]	Psoriasis: 118,178; Control: 3,262,337; Vitiligo: 79,907	OR of vitiligo in psoriasis 2.29 (1.56–3.37) OR of psoriasis in vitiligo 3.43 (1.86–6.33)
Fu (2018) [116]	Psoriasis: 212,544; Control: 7,581,541	OR of CD 1.7 (1.20–2.40); OR of UC 1.75 (1.49–2.05); RR of CD 2.74 (1.41–5.32); RR of UC 1.74 (0.72–4.17)
Alinaghi (2020) [117]	Prevalence of psoriasis in 199,672 patients with IBD; prevalence of IBD in 481,853 patients with psoriasis	Prevalence of psoriasis in CD 3.6% (3.1–4.6); in UC 2.8% (2.0–3.8) OR of psoriasis in IBD 1.8 (1.5–2.2); OR of psoriasis in CD 2.0 (1.4–2.9); OR of psoriasis in UC 1.5 (1.2–2.0); OR of CD in psoriasis 2.2 (1.6–3.1); OR of UC in psoriasis 1.6 (1.3–2.0).
Acharya (2020) [118]	Psoriasis: 136,536; Control: 5,716,558 Celiac disease: 48,803; Control: 1,833,038	OR of celiac disease in psoriasis 2.03 (1.49–2.75); OR of psoriasis in celiac disease 1.8 (1.36–2.38)
**PsA**		
Fu (2018) [116]	PsA:10,956; Control: 262,649	Case-control study: CD OR 2.20 (1.59–3.03); UC OR 1.91 (1.21–3.00); Cohort study: CD RR 2.74 (1.41–5.32); UC RR 1.74 (0.72–4.17)
Alinaghi (2020) [117]	Prevalence of IBD in 13,788 patients with PsA; prevalence of PsA in 47,740 patients with IBD	Prevalence of IBD 1.8% (1.0–2.9); prevalence of CD 1.2% (0.4–2.3); prevalence of UC 0.7% (0.3–1.2); prevalence of PsA in IBD 1.0% (0.4–1.9)
Acharya (2020) [118]	Celiac disease: 48,803; Control: 1,833,038	OR of PsA in celiac disease 2.31 (1.7–3.14)

Abbreviation: CD, Crohn’s disease; HR, hazard ratio; IBD, inflammatory bowel disease; MS, multiple sclerosis; OR, odds ratio; PsA, psoriatic arthritis; RR, relative risk; UC, ulcerative colitis. Values in brackets indicate 95% confidence intervals.

**Table 6:** Summary of recent (2010–2020) nationwide population-based cohort studies investigating the risk of psychiatric disorders in adult patients with psoriasis and psoriatic arthritis.

Psychiatric Disorder	Study	Study Population	Relative Risk of Measures
Psoriasis			
**Depression and/or suicidality**	Kurd (2010) [155]	Psoriasis: 149,998 (mild: 146,042; severe: 3,956) Control: 766,950	Depression HR in all psoriasis 1.39 (1.37–1.41); in mild psoriasis 1.38 (1.35–1.40); in severe psoriasis 1.72 (1.57–1.88) Suicidality HR in all psoriasis 1.44(1.32–1.57); in mild psoriasis 1.44 (1.32–1.57)
	Schmitt (2010) [156]	Psoriasis: 3,147 Control: 3,147	Depression OR 1.49 (1.20–1.86)
	Tsai (2011) [108]	Psoriasis: 51,800 Control: 207,200	Depression RR 1.50 (1.39–1.61)
	Kimball (2012) [151]	Pediatric patients with psoriasis: 7,404 Control: 37,020	Depression HR 1.23 (1.06–1.43)
	Parisi (2015) [10]	Psoriasis: 48,523 Control: 208,187	Depression HR 1.16 (1.01–1.34)
	Cohen (2016) [157]	History of psoriasis: 351 No history of psoriasis: 12,031	Major depression OR 2.09 (1.41–43.11); Symptoms of depression OR 1.44 (1.03–2.00)
	Jensen (2016) [158]	Mild psoriasis: 35,001, severe psoriasis: 7,510	Depression IRR in mild psoriasis 1.08 (1.04–1.12); in severe psoriasis 1.36 (1.27–1.46)
	Wu (2017) [159]	Psoriasis: 36,214 Control: 156,093	Depression IRR in psoriasis 1.14 (1.11–1.17)
	Tzur Bitan (2019) [160]	Psoriasis: 127,931 Control: 127,931	Depression OR 1.17 (1.08–1.26)
**Anxiety**	Kurd (2010) [155]	Psoriasis: 149,998 Control: 766,950	Anxiety HR in all psoriasis 1.31 (1.29–1.34); in mild psoriasis 1.29 (1.15–1.43); in severe psoriasis 1.29 (1.15–1.43)
	Kimball (2012) [151]	Pediatric patients with psoriasis: 7,404 Control: 37,020	Anxiety HR 1.32 (1.09–1.61)
	Tzur Bitan (2019) [160]	Psoriasis: 127,931 Control: 127,931	Anxiety OR 1.11 (1.01–1.23)
**Schizophrenia**	Tu (2017) [161]	Psoriasis: 10,796 Control: 10,796	Schizophrenia OR 1.44 (1.08– 1.92)
**Sleep disorder**	Tsai (2011) [108]	Psoriasis: 51,800 Control: 207,200	Sleep disorder RR 3.89 (2.26–6.71)
	Egeberg (2016) [162]	Psoriasis: 60,175 (mild: 53,290; severe: 6,885) Control: 5,393,040	Sleep apnea in mild psoriasis IRR 1.30 (1.17–1.44); severe psoriasis IRR 1.65 (1.23-2.22)
**PsA**			
**Depression and/or suicidality**	Wu (2017) [159]	PsA: 5,138 Control: 156,093	Depression IRR 1.22 (1.16–1.29)
**Sleep disorder**	Egeberg (2016) [162]	PsA: 6,348 Control: 5,393,040	Sleep apnea IRR 1.75 (1.35–2.26)

Abbreviations: HR, hazard ratio; IRR, incidence rate ratio; OR, odds ratio; PsA, psoriatic arthritis; RR, relative risk. Values in brackets indicate the 95% confidence interval.

## Abbreviations:

AIBD:	autoimmune bullous disease
BP:	bullous pemphigoid
CAD:	coronary artery disease
CD:	Crohn’s disease
CI:	confidence interval
CPAP:	continuous positive airway pressure
CV:	cardiovascular
CVD:	cardiovascular disease
HBV:	hepatitis B
HCV:	hepatitis C
HLA:	human leukocyte antigen
HR:	hazard ratio
IBD:	inflammatory bowel disease
IFN:	interferon
IHD:	ischemic heart disease
IL:	interleukin
IRR:	incidence rate ratio
MACE:	major cardiovascular events
MCP1:	monocyte chemoattractant protein
MI:	myocardial infarction
NETs:	neutrophil extracellular traps
NMSC:	nonmelanoma skin cancer
OR:	odds ratio
PsA:	psoriatic arthritis
PUVA:	psoralen and ultraviolet A
RR:	relative risk
SCC:	squamous cell carcinoma
SMR:	standardized mortality ratio
Th:	T helper
TNF:	tumor necrosis factor
UC:	ulcerative colitis
